# The impact of early use of statin in sepsis patients with acute kidney injury: a study based on MIMIC-IV

**DOI:** 10.3389/fphar.2025.1610450

**Published:** 2025-06-20

**Authors:** Xiaona Zhan, Haifeng Huang, Ling Ye, Shenglang Zhu, Jiehui Chen

**Affiliations:** Department of Nephrology, Shenzhen Nanshan People’s Hospital, Shenzhen, China

**Keywords:** sepsis, acute kidney injury, statin, 28-day mortality, MIMIC-IV

## Abstract

**Objective:**

Sepsis-associated acute kidney injury (SA-AKI) is a prevalent and serious condition in the ICU. The efficacy of statin in these patients remains unclear. The purpose of this study was to investigate the impact of the early use of statin on the prognosis of SA-AKI patients.

**Methods:**

The patients included in this study were derived from the MIMIC-IV database. Patients were divided into the non-statin and statin groups based on whether they received statin within 48 h of admission. After propensity score matching, time-dependent Cox regression analysis was used to evaluate the relationship between the early use of statin and mortality. The relationship between treatment and the recovery of renal function was assessed by logistic analysis, and the association between the use of statin and length of ICU stay was evaluated by the negative binomial regression model.

**Results:**

A total of 11,667 patients were included in the study. After propensity score matching, there were 1,585 patients in each group, and the baselines between the two groups were comparable. Although statin use was associated with prolonged ICU stay, it was significantly linked to reduced 28-day and 90-day mortality and improved renal function recovery. Subgroup analysis indicated that the protective effect of early statin use was more effective in patients with hypertension. In comparison with the non-statin group, the use of different types of statin were associated with reduced 28-day mortality in sepsis patients with AKI. Compared with the rosuvastatin group, there was no significant difference in reducing the risk of mortality among other types of statins.

**Conclusion:**

In sepsis patients with AKI, the early use of statin was associated with a reduction in 28-day and 90-day mortality. The early use of statin plays a protective role in patients with SA-AKI.

## 1 Introduction

Sepsis is defined as infection-induced systemic inflammatory response syndrome, which could result in life-threatening organ dysfunction due to the imbalance of hemodynamics, inflammatory mediators, and immune responses (Cecconi et al., 2018; Cajander et al., 2024). The kidney is one of the most frequently affected organs in sepsis patients. Sepsis-associated acute kidney injury (SA-AKI) occurs in a significant proportion of septic patients, with reported incidence rates widely ranging from 14% to 87% (Zarbock et al., 2023). SA-AKI is defined as AKI occurring within 7 days of a sepsis diagnosis (Kellum and Lameire, 2013; Zarbock et al., 2023), and carries a poor prognosis. Beyond high mortality rates (11%–77%), SA-AKI can lead to chronic kidney disease, severely impacting long-term health and quality of life (Poston and Koyner, 2019). Current treatments for SA-AKI, including fluid resuscitation, renal replacement therapy, and antibiotic therapy, have not sufficiently reduced its high mortality rates (Peerapornratana et al., 2019), highlighting the need for novel therapeutic strategies.

Statin, primarily recognized for its inhibition of HMG-CoA reductase to reduce cholesterol levels and decrease cardiovascular events (Chou et al., 2022), also exhibits pleiotropic effects, including anti-inflammatory, immunomodulatory, and antioxidant properties (Satny et al., 2021; Kansal et al., 2023; Mollazadeh et al., 2021). These non-lipid-lowering effects have prompted investigation into its role in other conditions. Multiple reports have suggested a protective effect of statin in preventing contrast-induced AKI and post-cardiac surgery AKI (Anjum et al., 2019; Mikhailidis and Athyros, 2013; Putzu et al., 2016; Bellomo, 2016).

However, the efficacy of statin in the specific context of SA-AKI remains controversial. For instance, a recent study indicated that pre-ICU statin use could reduce AKI risk in obese septic patients (Xiong and Liu, 2025), while another found no significant prognostic improvement in statin-treated sepsis patients with severe AKI (Wang et al., 2019). Given these conflicting findings, the precise impact of statin on SA-AKI outcomes and the underlying mechanisms necessitate further exploration.

This study aimed to investigate the effect of early statin administration (within 48 h of ICU admission) on mortality, renal function recovery, and length of ICU stay in patients with SA-AKI. We hypothesized that early statin use might provide a protective benefit in SA-AKI patients.

## 2 Methods

### 2.1 Data sources

The data source of this study is the MIMIC-IV 3.1 database (Johnson et al., 2023), which was updated on 11 October 2024. The database contains information about more than 65,000 hospitalized patients at Beth Israel Deaconess Medical Center in Boston, United States of America. Research author Zhan has obtained the access and rights to use the database. It is worth noting that the MIMIC database has been approved by the Massachusetts Institute of Technology (MIT) institutional review board, thus this study can be exempted from the additional institutional IRB approval process.

### 2.2 Patients included in the study

**FIGURE 1:**
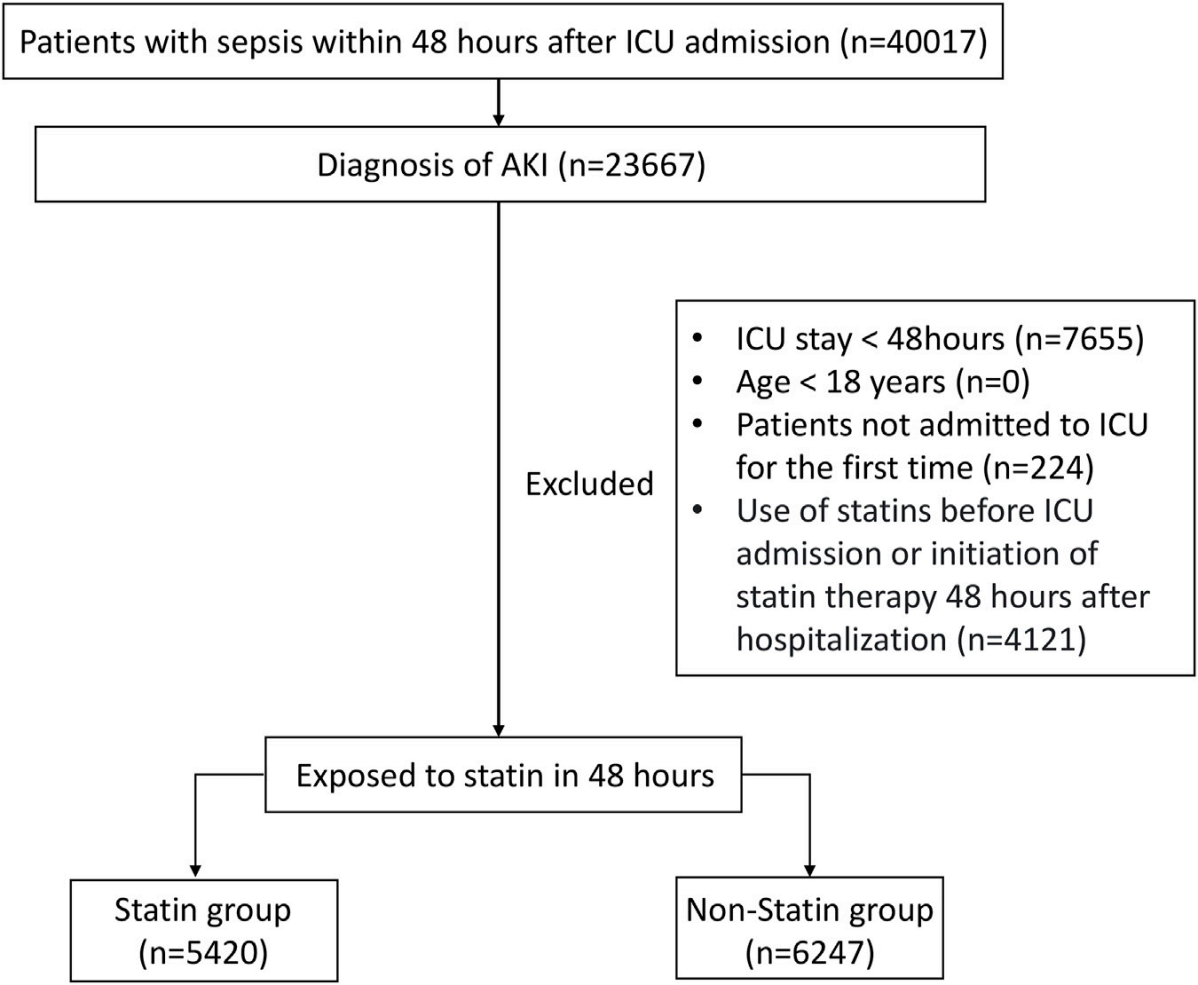
Flow chart of the study selection process.

The target population of this study was sepsis patients with AKI who had been hospitalized in the intensive care unit (ICU) for more than 48 h. The specific inclusion criteria were as follows: (1) Patients with sepsis within 48 h after being admitted to ICU; (2) Patients with AKI occurring up to 48 h after sepsis happened (Zarbock et al., 2023); (3) Age ≥18 years; (4) Patients admitted to the ICU for the first time with a length of stay of more than 48 h in ICU. For patients who had been admitted to the ICU multiple times, only the first ICU record was selected and included in the study. The definition of sepsis was based on the criteria of Sepsis 3.0: meeting the infection indicators combined with an increase of two points or more in SOFA score or having organ dysfunction caused by infection (Singer et al., 2016). The definition of AKI adopted the KDIGO standard: an increase in serum creatinine by ≥ 0.3 mg/dL within 48 h, a serum creatinine level rising to ≥1.5 times the baseline value within 7 days, or a urine output of <0.5 mL/kg/h for 6 h (Kellum and Lameire, 2013). According to whether the patients used statin within 48 h after being admitted to the ICU, the patients were divided into the statin group and the non-statin group. The process diagram was shown in Figure 1.

### 2.3 Variables

The following data was collected: (1) General information: gender, age, weight, clinical outcome; (2) Laboratory indicators: white blood cell, hemoglobin, platelet, serum creatinine, urea nitrogen, liver function, arterial blood gas; (3) Disease severity: SOFA score and GCS score; (4) Vital signs: heart rate, mean blood pressure, urine output on first day; (5) Comorbidity: heart failure, myocardial infarction, diabetes, pulmonary disease, hypertension, chronic kidney disease; (6) Treatment after admission to the ICU (within 24 h): use of renal replacement therapy, mechanical ventilation, vasoactive drugs, and colloids. Vasopressor drugs include: dopamine, epinephrine, norepinephrine, phenylephrine, vasopressin, dobutamine, and milrinone. Colloids include albumin and plasma.

The above data was extracted by executing Structured Query Language (SQL) using Navicat Premium 17 software. For laboratory indicators measured multiple times within 24 h of admission, representative maximum or minimum values were selected based on clinical significance, such as the highest partial pressure of carbon dioxide (PCO2), lowest pH, lowest pressure of oxygen (PO2), and bicarbonate concentration (BC).

### 2.4 Outcomes

The primary outcome was 28-day mortality, and the secondary outcomes included 90-day mortality, recovery of renal function, and the length of ICU stay. Recovery of renal function required the following two conditions to be met: (1) The urine output on discharge >0.5 mL/kg/h for 24 h; (2) A return to a creatinine level of 150% of the baseline on ICU discharge (Zhao et al., 2020).

### 2.5 Statistical analysis

For categorical data such as gender and race, the Chi-square test was used for group comparison, and the results were expressed as frequencies and percentages (%). For measurement data such as age and baseline creatinine values, the Shapiro-Wilk (S-W) test was first used to check whether they conformed to a normal distribution. Measurement data that conformed to a normal distribution were expressed as mean ± standard deviation, and the independent-samples t-test was used for group comparison. Measurement data that did not conform to a normal distribution were expressed as median and interquartile range, and the Mann-Whitney U test was used for group comparison.

To balance the baseline between statin and non-statin groups, nearest neighbor matching was used for propensity score matching (PSM) with a caliper width of 0.02 logits. Covariate balance was assessed both before and after PSM using the standardized mean difference (SMD). It was deemed that the variables were balanced between groups when the SMD is less than 0.1 (Zhang, 2017). Missing values in the data were handled using the multiple imputation method. Time-dependent Cox regression analysis was used to assess the association between the early use of statin and mortality in sepsis patients with AKI. Logistic regression analysis was used to evaluate the relationship between the treatment and renal recovery. Negative binomial regression model was used to assess the association between the use of statin and length of ICU stay. The Kaplan-Meier curve described the survival probabilities of patients, and the log-rank test was used to calculate inter-group differences. Subgroup analysis based on baseline characteristics such as age, AKI stage, and SOFA score of the two groups was used to evaluate the presence of interaction. The statistical data in this study were analyzed by R (4.4.2). Bilateral p-values <0.05 are considered statistically significant.

## 3 Results

### 3.1 Baseline characteristics

A total of 23,667 patients with SA-AKI were included in this study, of whom 11,667 met the established inclusion criteria (Figure 1). Based on whether statin was used within 48 h after ICU admission, the patients were divided into a statin group and a non-statin group. The baseline characteristics of patients in the two groups are detailed in Table 1. Compared with the non-statin group, the statin group had an older median age, heavier weight, and a higher proportion of men. The proportion of patients with heart failure, myocardial infarction, diabetes, or hypertension was higher in the statin group. The level of AST, ALT in non-statin group was higher than that of statin group. The lactate level of patients in the non-statin group was slightly higher, and the bicarbonate level and pH value were slightly lower. For therapeutic intervention, the number of patients who received vasoactive drugs or mechanical ventilation on the first day of ICU admission was higher in the statin group. However, there was no significant difference between the two groups in the proportion of patients receiving renal replacement therapy or using colloid on the first day. In addition, patients in the statin group had a higher urine output than those in the non-statin group on the first day.

**TABLE 1:** Baseline characteristics of patients before PSM.

Characteristic	Non-statin	Statin
	N = 6,247	N = 5,420
Age	62.13 [50.00, 75.44]	72.77 [64.16, 80.83]
Male (%)	3,550 (56.8)	3,273 (60.4)
Race (%)		
Asian	165 (2.6)	121 (2.2)
Black	508 (8.1)	572 (10.6)
Other	1834 (29.4)	1,071 (19.8)
White	3,740 (59.9)	3,656 (67.5)
Weight	79.60 [66.00, 97.00]	81.90 [69.00, 97.23]
SOFA score	6.00 [4.00, 10.00]	6.00 [4.00, 8.00]
GCS score	15.00 [13.00, 15.00]	15.00 [13.00, 15.00]
MBP	75.75 [69.94, 82.96]	74.58 [69.47, 80.80]
Heart Rate	89.22 [77.61, 101.89]	82.80 [73.91, 93.43]
AKI stage (%)		
1	5,493 (87.9)	4,868 (89.8)
2	533 (8.5)	388 (7.2)
3	221 (3.5)	164 (3.0)
Heart failure (%)	1,394 (22.3)	2,715 (50.1)
Pulmonary disease (%)	1,480 (23.7)	1,665 (30.7)
Diabetes (%)	1,263 (20.2)	2,485 (45.8)
Myocardial infarction (%)	386 (6.2)	1865 (34.4)
Hypertension (%)	2,234 (35.8)	2,154 (39.7)
CKD (%)	116 (1.9)	229 (4.2)
White blood cell	11.95 [8.28, 17.10]	11.90 [8.60, 16.30]
Platelet	188.00 [126.00, 263.00]	190.00 [143.00, 256.00]
Hb	10.50 [8.80, 12.20]	10.10 [8.60, 11.90]
Scr	1.10 [0.70, 1.70]	1.20 [0.80, 1.90]
Bun	22.00 [14.00, 37.00]	24.00 [16.00, 39.75]
ALT	29.00 [16.00, 66.75]	23.00 [14.00, 43.00]
AST	52.00 [28.00, 132.00]	36.00 [23.00, 75.00]
PO_2_	61.00 [41.00, 92.00]	66.00 [39.00, 94.00]
PCO_2_	47.00 [40.00, 56.00]	48.00 [42.00, 56.00]
PH	7.30 [7.22, 7.37]	7.31 [7.25, 7.36]
Bicarbonate	22.00 [19.00, 25.00]	23.00 [20.00, 25.00]
Lactate	2.30 [1.50, 3.30]	2.20 [1.50, 3.10]
RRT (%)	552 (8.8)	483 (8.9)
Ventilation (%)	4,823 (77.2)	4,497 (83.0)
Vasoactive drug (%)	2,596 (41.6)	2,792 (51.5)
Colloid use (%)	1,499 (24.0)	1,237 (22.8)
Urine output	1200.00 [710.00, 1910.00]	1315.00 [795.00, 1975.00]

Abbreviations: PSM, propensity score matching; MBP, mean blood pressure; AKI, acute kidney injury; CKD, chronic kidney disease; Hb, hemoglobin; Scr, Serum creatinine; BUN, blood urea nitrogen; ALT, aminotransferase; AST, aspartate Aminotransferase; RRT, renal replacement therapy.

To further balance the baseline characteristics of the two groups, PSM was performed in this study, and the baseline characteristics of the statin group and the non-statin group after matching are shown in Table 1. After PSM, the SMD of the covariates in both groups was less than 0.1 (Supplementary Figure S1), indicating that the baseline of the two groups had a roughly good balance after matching.

### 3.2 Association between statin and mortality outcomes

The research results indicated that, after PSM, early oral administration of statin was significantly correlated with reduced 28-day mortality (HR 0.59; 95% CI 0.51–0.67; P < 0.001) and 90-day mortality rates in patients with SA-AKI (HR 0.64; 95% CI 0.57–0.73; P < 0.001) (Table 2). The results of the analysis after PSM were consistent with those before PSM (Supplementary Table S1). This finding suggested that early oral administration of statin had a positive impact on reducing the short-term mortality risk in sepsis patients with AKI.

**TABLE 2:** Association between use of statin and mortality in sepsis patients with AKI (after PSM).

Outcome	Non-statin	Statin	HR (95% CI)	p-value
28-day mortality, n (%)^a^	542 (34.20%)	322 (20.32%)	0.59 (0.51, 0.67)	<0.001
90-day mortality, n (%)^b^	659 (41.58%)	441 (27.82%)	0.64 (0.57, 0.73)	<0.001

AKI, acute kidney injury; HR, hazard ratio; CI, confidence interval.

^a^ Adjusted variables included age, SOFA, score, white blood cell, BUN, ALT, AST, PH, bicarbonate, lactate, weight, heart failure, myocardial infarction, urine output, use of colloid, and use of renal replacement therapy.

^b^ Adjusted variables included age, weight, SOFA, score, white blood cell, BUN, ALT, AST, lactate; PH, bicarbonate, hypertension, heart failure, myocardial infarction, urine output, use of colloid, and use of renal replacement therapy.

### 3.3 Association between statin and recovery of renal function

Our study further explored the effect of early statin use on the recovery of renal function in patients with SA-AKI. In the cohort after PSM, after adjusting for multiple confounding factors, the use of statin was associated with the recovery of renal function (OR 1.17; 95% CI 1.02–1.37; P < 0.047) (Table 3). Statin promoted the recovery of renal function in sepsis patients with AKI.

**TABLE 3:** Association between use of statin and recovery of renal function in sepsis patients with AKI (after PSM).

Outcome	Non-statin	Statin	Or (95% CI)	p-value
Recovery of renal function, n (%)	880 (55.52%)	913 (57.60%)	1.17 (1.02, 1.37)	0.047

AKI, acute kidney injury; OR, odds ratio; CI, confidence interval.

Adjusted variables included age, gender, weight, SOFA, score, heart failure, BUN, ALT, AST, lactate; PH, hypertension, diabetes, myocardial infarction, urine output, and use of renal replacement therapy.

### 3.4 Association between statin and length of ICU stay

Regarding the length of ICU stay, the analysis after PSM showed that the use of statin was associated with prolonged ICU stay (IRR 1.062; 95% CI 1.004–1.123; P = 0.036) (Table 4).

**TABLE 4:** Association between use of statin and length of ICU stay in sepsis patients with AKI.

Cohort	IRR	95% CI	p-value
Before PSM^a^	1.043	(1.002, 1.085)	<0.040
After PSM^b^	1.062	(1.004, 1.123)	0.036

AKI, acute kidney injury; IRR, incidence rate ratio; CI, confidence interval.

^a^ Adjusted variables included age, gender, race, SOFA, score, mean heart rate, white blood cell; AST, lactate; PH, bicarbonate, urine output, weight, chronic kidney disease, hypertension, use of renal replacement treatment.

^b^ Adjusted variables included age, gender, race, SOFA, score, mean heart rate, BUN, AST, lactate; PH, urine output, chronic kidney disease, myocardial infarction, use of renal replacement treatment.

### 3.5 Association between different types of statin and mortality

**FIGURE 2:**
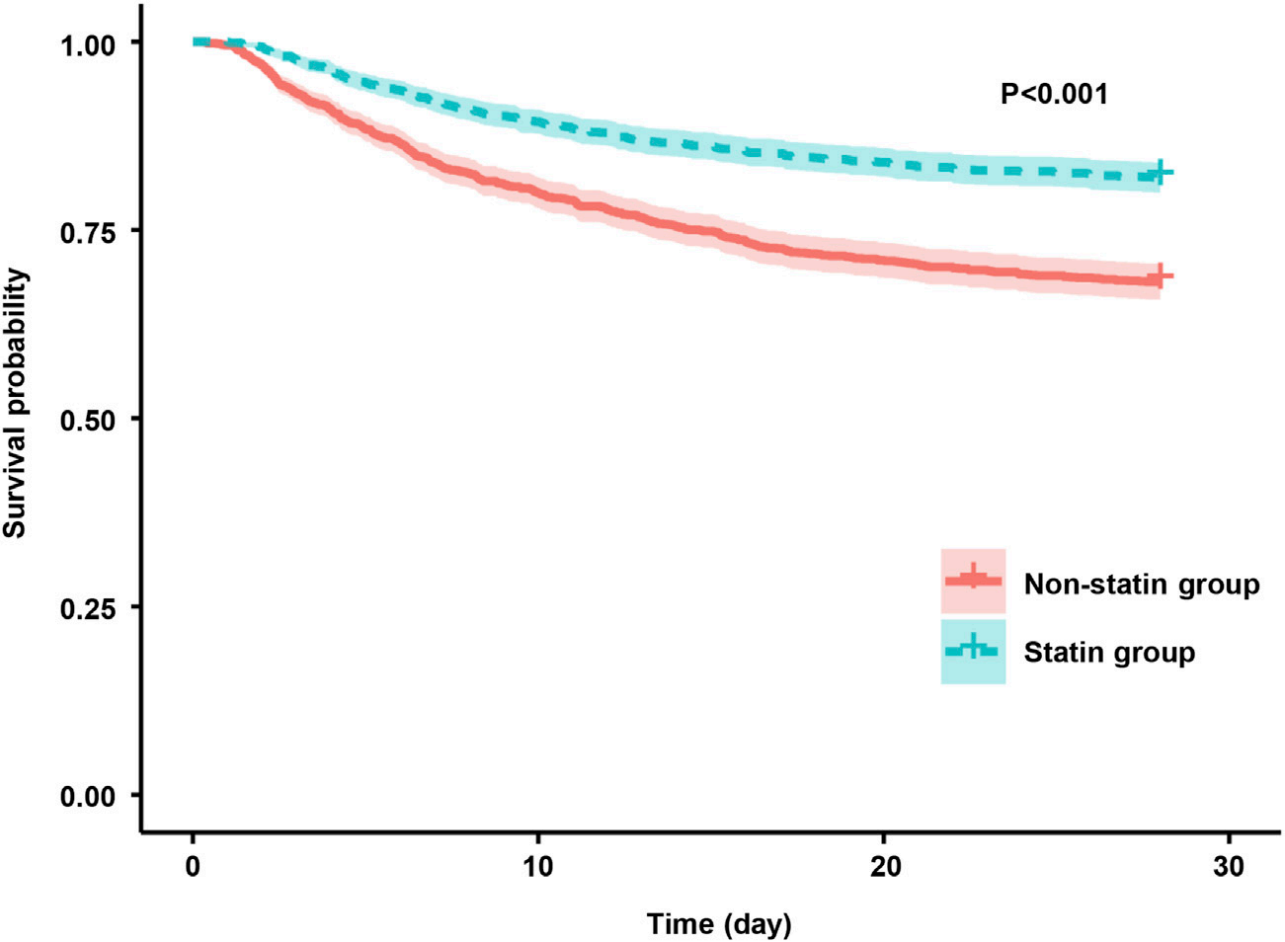
Kaplan-Meier survival curves for the 28-day mortality risk for the sepsis patients with AKI in the statin group and non-statin group.

**FIGURE 3:**
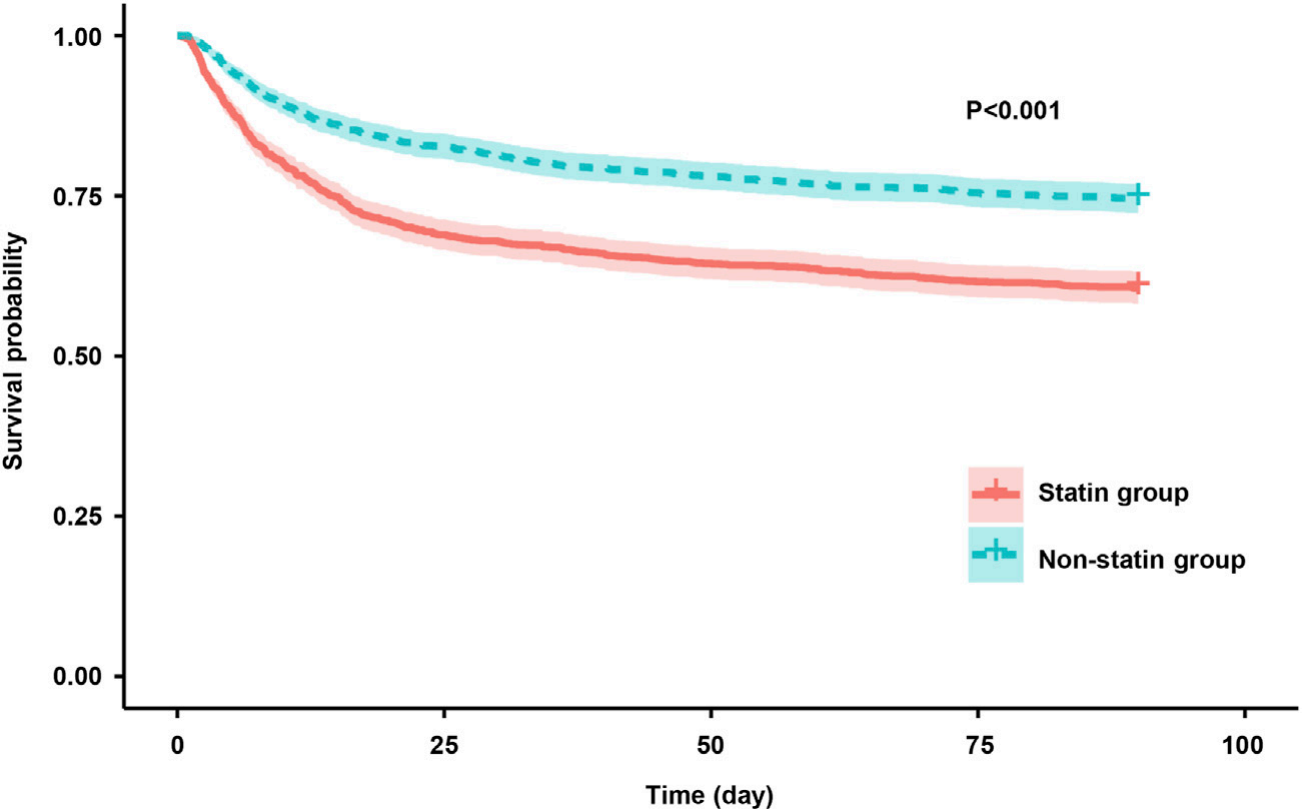
Kaplan-Meier survival curves for the 90-day mortality risk for the sepsis patients with AKI in the statin group and non-statin group.

The results of the Kaplan-Meier analysis showed that the 28-day and 90-day survival probability of patients receiving statin was higher than that of patients not receiving statin (log-rank test: P < 0.001) (Figures 2, 3). This study further investigated the impact of different types of statin on the primary outcomes. Given that this study focused on the effect of monotherapy, patients using multiple statins were not included in the study. Meanwhile, the lovastatin group was also excluded due to the insufficient sample size. The analysis showed that the use of atorvastatin (HR 0.61; 95% CI 0.52–0.72; P < 0.001), pravastatin (HR 0.59; 95% CI 0.37–0.92; P = 0.019), rosuvastatin (HR 0.63; 95% CI 0.41–0.95; P = 0.029), and simvastatin (HR 0.51; 95% CI 0.39–0.69; P < 0.001) was associated with lower mortality in patients with SA-AKI compared with patients without statin (Table 5). Compared with rosuvastatin, there was no statistically significant difference in the reduction of 28-day mortality among atorvastatin (HR 1.03; CI 0.63–1.51; P = 0.89), pravastatin (HR 1.06; CI 0.52–1.74; P = 0.86), and simvastatin (HR 1.16; CI 0.52–1.42; P = 0.55) (Table 6).

**TABLE 5:** Association between different types of statin and 28-day mortality in patients (Non-statin as reference).

Variables	28-day mortality, n (%)	HR (95% CI)	p-value
Non-statin	575 (33.3%)	1 (reference)	1 (reference)
Atorvastatin	213 (21.4%)	0.61 (0.52, 0.72)	<0.001
Pravastatin	20 (19.4%)	0.59 (0.37, 0.92)	0.019
Rosuvastatin	23 (22.8%)	0.63 (0.41, 0.95)	0.029
Simvastatin	51 (16.7%)	0.51 (0.39, 0.69)	<0.001

AKI, acute kidney injury; HR, hazard ratio; CI, confidence interval.

Adjusted variables included age, weight, SOFA, score, white blood cell, Hb, BUN, ALT, AST, lactate; PH, bicarbonate, hypertension, heart failure, myocardial infarction, urine output, use of colloid, use of renal replacement treatment.

**TABLE 6:** Association between different types of statin and 28-day mortality in patients (Rosuvastatin group as reference).

Variables	28-day mortality, n (%)	HR (95% CI)	p-value
Rosuvastatin	23 (22.8%)	1 (reference)	1 (reference)
Atorvastatin	185 (21.4%)	1.03 (0.63, 1.51)	0.89
Pravastatin	20 (19.4%)	1.06 (0.52, 1.74)	0.86
Simvastatin	51 (16.7%)	1.16 (0.52, 1.42)	0.55

AKI, acute kidney injury; HR, hazard ratio; CI, confidence interval.

Adjusted variables included age, weight, SOFA, score, white blood cell, Hb, BUN, ALT, AST, lactate; PH, bicarbonate, hypertension, heart failure, myocardial infarction, urine output, use of colloid, use of renal replacement treatment.

### 3.6 Subgroup analysis

**FIGURE 4:**
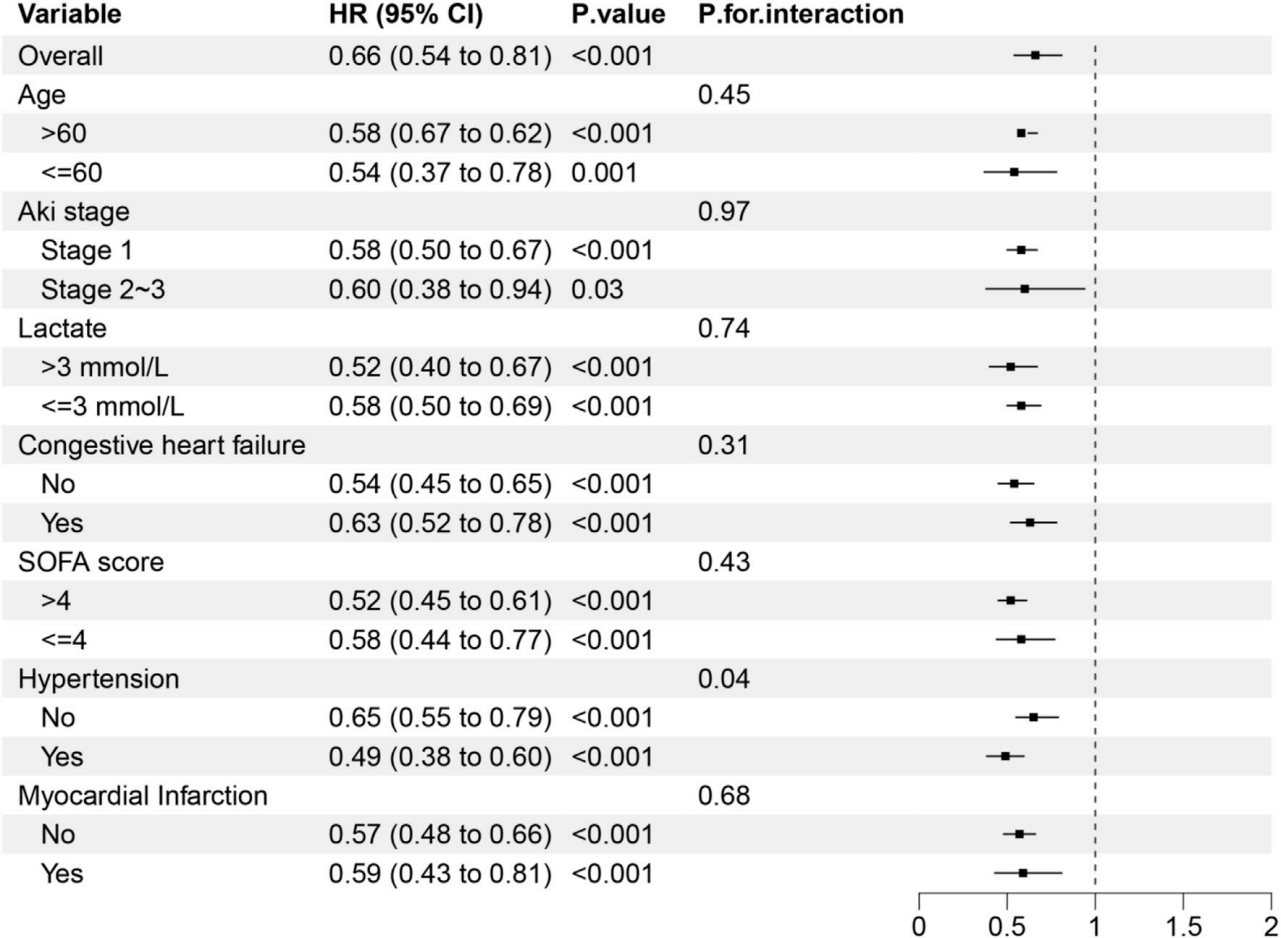
Subgroup analysis of the relationship between statin use and 28-day mortality.

As shown in Figure 4, the protective effect of early statin therapy was more pronounced in patients with hypertension. No significant interaction was found between the early use of statin and other subgroups (P for heterogeneity >0.05 in subgroups of age, AKI stage, lactate level, heart failure, and SOFA score).

## 4 Discussion

This retrospective study, based on the MIMIC-IV database, demonstrated that early statin use was associated with reduced short-term mortality and earlier recovery of renal function in patients with SA-AKI. However, our research indicated that statin treatment was associated with prolonged ICU stay.

Recent literature on the role of statin in patients with acute kidney injury (AKI) presents conflicting conclusions. A retrospective study demonstrated that statin use was associated with reduced in-hospital and cardiovascular-related mortality; however, no significant association was found between statin use and sepsis-related mortality (Zheng et al., 2024). Research focusing on sepsis patients has also yielded divergent results. Xiong and Liu (2025) reported that pre-ICU statin use decreased the risk of AKI in sepsis patients with a body mass index (BMI) greater than 30 kg/m^2^. In contrast, Wang et al. (2019) found that statin therapy was associated with higher 90-day mortality in sepsis patients requiring dialysis. Furthermore, Murugan et al. (2012) showed that hospitalized pneumonia patients who continued statin use had an increased risk of AKI. Notably, none of these previous studies specifically targeted the sepsis-associated acute kidney injury (SA-AKI) subgroup, leaving the impact of statin on the prognosis of SA-AKI patients largely undetermined. Our study fills this research gap by demonstrating that early statin use in patients with SA-AKI was associated with reduced 28-day and 90-day mortality, enhanced renal function recovery. However, this study found that statin use was associated with prolonged ICU stay. Patients in the ICU using statin may have more severe conditions, underlying diseases, and complications. Even if the drugs do not directly cause adverse consequences, these patients still require longer treatment and observation, thereby leading to a prolongation of ICU stay.

Basic research has investigated the protective mechanisms of statin in SA-AKI. In a lipopolysaccharide (LPS)-induced murine acute kidney injury model, simvastatin alleviated tubular necrosis and hypoxia by inhibiting the elevation of tumor necrosis factor-α (TNF-α) (Yasuda et al., 2006). Another animal study on SA-AKI showed that simvastatin was shown to improve renal function in mice by regulating anti-apoptotic molecules (Nežić et al., 2020). Based on these prior studies, we hypothesize that statin may improve SA-AKI through these mechanisms, thereby reducing short-term mortality and promoting renal recovery.

The influence of different statin types on patient prognosis represents an important research domain. For example, a multicenter randomized trial in diabetic patients revealed no significant differences in urine protein-creatinine ratio (UPCR) changes between atorvastatin and rosuvastatin; however, the rosuvastatin group exhibited a significant decline in estimated glomerular filtration rate (eGFR), along with higher incidences of acute renal failure and doubling of serum creatinine levels (de Zeeuw et al., 2015). In our SA-AKI cohort, patients receiving any statin demonstrated a lower short-term mortality than the non-statin group. Atorvastatin, simvastatin, or pravastatin did not significantly reduce 28-day mortality when compared with rosuvastatin. Given the focus of this study on 28-day mortality, additional clinical trials are warranted to fully evaluate the long-term effects of different statin types.

In this study, the results of subgroup analysis showed that the protective effect of early use of statin was more significant in hypertension patients. This phenomenon may be attributed to the fact that patients with hypertension may have a medication history of RAS inhibitors (such as ACEI/ARB). These agents may exert synergistic renoprotective effects in combination with statin by reducing proteinuria and inhibiting renal interstitial fibrosis. In patients without hypertension, the relatively intact endothelial function might limit the additional renoprotective benefits of statin.

The safety of statin use in critically ill patients requires attention. Previous RCT results have shown that critically ill COVID-19 patients treated with simvastatin had a higher incidence of elevated liver enzymes and creatine kinase levels (Remap-Cap, 2023). However, a meta-analysis on the efficacy of statin in sepsis patients showed no statistically significant difference in the incidence of liver injury or myopathy between the statin and placebo group (Cai et al., 2021). While current research findings on the safety of statin use in critically ill patients are inconsistent, it is recommended to closely monitor liver function and creatine kinase levels during statin treatment.

This study has several limitations inherent in its retrospective design. First, there are unknown or unmeasured confounding factors, including indication confounding. Patients who initiated statin therapy may have received closer monitoring and care due to preexisting cardiovascular diseases. In contrast, patients who did not use statin may have had contraindications such as hepatic or renal dysfunction, and the short-term mortality of this group may be influenced by factors such as hepatic/renal insufficiency or disease severity. Although liver function data were collected and PSM was applied to adjust for intergroup disparities, and multivariate regression models with subgroup analyses were used to minimize confounding effects, residual confounding bias may persist. Second, this study cannot fully trace whether patients had a history of statin use before admission. This may lead to inaccurate grouping, and the estimation of the effect of statin may be biased, affecting the analysis of the association between statin and clinical outcomes. Besides, this study cannot comprehensively assess whether patients had complications such as statin-related liver injury or rhabdomyolysis. The safety of statin in sepsis patients with AKI remains unclear.

In summary, this retrospective study based on the MIMIC-IV database indicated that early use of statin reduced short-term mortality in patients with SA-AKI. More clinical trials are needed in the future to confirm the efficacy and safety of the early use of statin in SA-AKI.

## Data Availability

The raw data supporting the conclusions of this article will be made available by the authors, without undue reservation.
