# Supplementary material for: The impact of early use of statin in sepsis patients with acute kidney injury: a study based on MIMIC-IV
Source: Front Pharmacol. 2025 Jun 20;16:1610450. doi: 10.3389/fphar.2025.1610450 (PMC12226583; doi:10.3389/fphar.2025.1610450)
Supplement: Supplementary file 1 [file Table1.DOCX]

Table S1. Association between use of statin and mortality in sepsis patients with AKI (before PSM)

| Outcome | Non-statin | Statin | HR  (95% CI) | p-value |
| --- | --- | --- | --- | --- |
| 28-day mortality, n (%) ^a^ | 1992 (31.89%) | 1093 (20.17%) | 0.56 (0.50, 0.62) | < 0.001 |
| 90-day mortality, n (%) ^b^ | 2415 (38.66%) | 1515 (27.95%) | 0.60 (0.54, 0.66) | < 0.001 |

AKI, acute kidney injury; HR, hazard ratio; CI, confidence interval.

^a^ Multivariate Cox proportional hazard models were used by adjusting for ‌age, race, weight, SOFA score, GCS score, AKI stage, heart rate, mean blood pressure, white blood cell, HB, SCR, BUN, ALT, AST, lactate, PO_2_, PH, bicarbonate, hypertension, heart failure, myocardial infarction, urine output, use of ventilation, use of vasoactive drug, use of renal replacement therapy.

^b^ Multivariate Cox proportional hazard models were used by adjusting for ‌age, race, weight, SOFA score, GCS score, AKI stage, heart rate, mean blood pressure, white blood cell, HB, SCR, BUN, ALT, AST, lactate, PO_2_, PH, bicarbonate, hypertension, heart failure, myocardial infarction, use of ventilation, use of vasoactive drug, use of renal replacement therapy.
