# Supplementary figures and images for: The impact of early use of statin in sepsis patients with acute kidney injury: a study based on MIMIC-IV
Source: Front Pharmacol. 2025 Jun 20;16:1610450. doi: 10.3389/fphar.2025.1610450 (PMC12226583; doi:10.3389/fphar.2025.1610450)

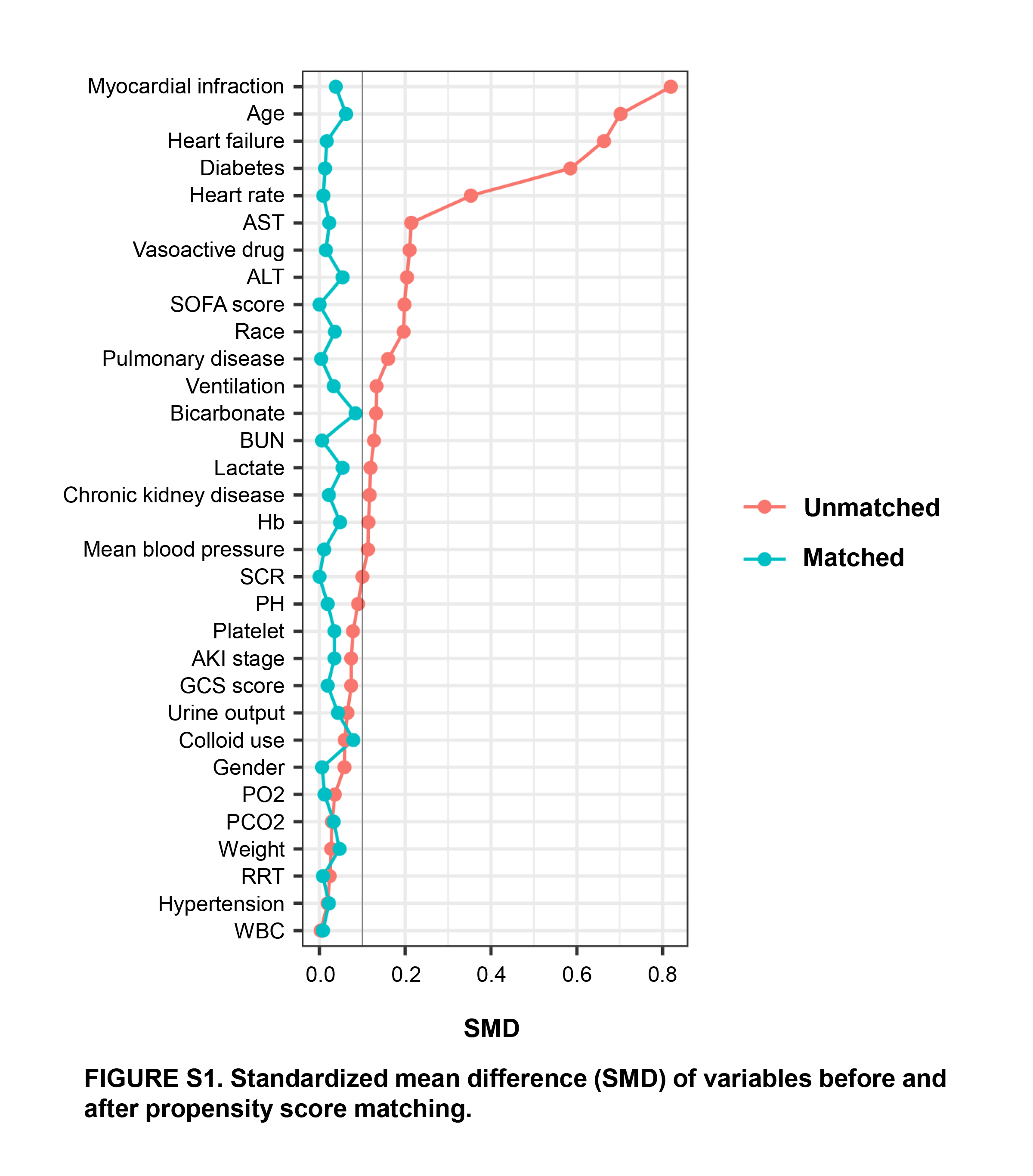

Supplement: Supplementary file 2 [file Image1.tif]
