# Supplementary material for: The impact of early use of statin in sepsis patients with acute kidney injury: a study based on MIMIC-IV
Source: Front Pharmacol. 2025 Jun 20;16:1610450. doi: 10.3389/fphar.2025.1610450 (PMC12226583; doi:10.3389/fphar.2025.1610450)
Supplement: Supplementary file 3 [file Table2.docx]

Table S2. Association between use of statin and recovery of renal function in sepsis patients with AKI (before PSM)

| Outcome | Non-statin | Statin | OR  (95% CI) | p-value |
| --- | --- | --- | --- | --- |
| Recovery of renal function, n (%) ^c^ | 3332 (60.1%) | 2976 (62.5%) | 1.14 (1.01, 1.29) | 0.045 |

AKI, acute kidney injury; OR, odds ratio; CI, confidence interval.

Multivariate logistic regression models were used by adjusting gender, weight, SOFA score, AKI stage, heart rate, mean blood pressure, platelet, HB, SCR, ALT, AST, lactate, PO_2_, PH, bicarbonate, heart failure, myocardial infarction, diabetes, hypertension, use of colloid, use of vasoactive drug, use of renal replacement therapy.
